# Abstract of Paper on Operation for Closure of the Hard Palate and Hare-Lip Immediately after Birth

**Published:** 1882-10

**Authors:** D. H. Goodwillie


### SELECTIONS.

### ABSTRACT OF PAPER ON OPERATION FOR CLOSURE OF THE HARD PALATE AND HARE-LIP IMMEDIATELY AFTER BIRTH.

In many cases there is tissue enough developed, but there is a failure to unite, and the maxillary bones are separated, making the diameter from side to side greater in proportion to other parts of the face. What is of special importance in this method is to restore the bones to the normal position without any loss of hard or soft tissue, except so much as would be required to freshen the edges of opposing parts. The cleft of the hard palate and lip, if any exist, should be done soon after birth, and before the child is two months old, to avoid injuring the developing teeth. The closure of the soft palate, if it is to be by a surgical operation, should be done, if possible, before the child begins to speak, at about 2 or 3 years of age.

My method I will illustrate by a case represented in the wax model that I pass around, and which was taken from a cast of a child one week old, and also by diagrams. By its examination you will see presented a cleft of the lip on the left side, and also a cleft through the left of the intermaxillary, extending through hard and soft palate. Bone development has been sufficient in amount, but there being a failure to unite the two sides in the process of growth, the bone became separated, and the intermaxillary attached to the right maxillary leaves the normal anatomical form of the anterior alveolus and becomes more or less straight, the end of the intermaxillary protrudes forward into the cleft of the lip. By this straightening process the nose is carried to the right side, as the anterior part of the nasal septum rests on the intermaxillary, while the left ala is very much stretched to the left. The usual practice is to only close the lip in infancy ; but in order to do so it is necessary to have the protruding end of the intermaxillary removed either by cutting it away or crushing it, both of which means is bad surgery. In the former, the bone is removed with all the teeth germs ; and in the latter, the germs are destroyed and the parts misshapen. And also to dissect the lips from the bone to allow them to approximate.

The operation for the relief of the deformity the model represents in this case was made in the following manner : The child was placed under an anæsthetic, and by means of a small revolving knife and the surgical engine a small V-shaped section was removed inside of the alveolar process of the intermaxillary, also running up into the septum a very little, and at the same time the edges of cleft of the hard palate was freshened by the revolving knife. Holes were also cut on either side of the hard palate for the purpose of passing suture pin clamps to hold the maxillæ together. Just enough was taken away by the V-shaped section to allow the alveolus of the intermaxillary to resume its normal position. Now, by means of a properly-constructed forceps the maxillary bones were forced together so as to close the cleft in the hard palate. Then a nasal forcep was passed into the nostrils, grasping the septum, and the nose drawn into perpendicular position, and, at the same time, the intermaxillary was forced into its normal place, closing up the V-shaped section made by the revolving knife. The alveolar ridge of the intermaxillary now connects with the maxillary of the opposite side. They are now held together by the suture pin clamps which I have devised for the purpose, and made of the very best steel and gold plated. The cleft in the lip is now closed by first carefully applying the compression lip clamp on each side of the cleft lip to prevent hemorrhage. After the edges are pared, then carefully approximate both skin and mucous membrane by passing the first suture in the vestibule of the nostril and ending with the vermilion border, and then complete the operation by passing the suture kin clamps to take the stress off the sutures.

In all simple or double clefts, all bone tissue should be preserved to prevent deformity in adult life.

The advantages of this method are : 1st, The cleft in the hard palate is closed in all cases where there is the normal amount of bone developed. 2d, The alveolar ridge with the tooth germs are saved and brought into place, securing, as near as possible, the normal outline of the mouth and subsequent development of the teeth. 3d, The nose is brought into normal position and over-distended nostril restored. 4th, The external normal appearance of the face is reclaimed.—*D. H. Goodwillie in Canada Medical and Surgical Jour.*

